# Supplementary material for: Cells of the adult human heart
Source: Nature. 2020 Sep 24;588(7838):466–72. doi: 10.1038/s41586-020-2797-4 (PMC7681775; doi:10.1038/s41586-020-2797-4)
Supplement: Supplementary file 1 — FACS gating strategy for removal of cell debris and purification of nuclei. a. Size gating to remove doublets and aggregates was applied (SSC-A, FSC-A, SSC-W, SSC-H, FSC-H, FSC-W), followed by sorts for Hoechst-positive nuclei (purple population). Used for all nuclei processed in North America b. Example of negative control (no NucBlue Live ReadyProbes Reagent applied, ThermoFisher) with the same parameters as a. c. Gating strategy used for all nuclei processed in the United Kingdom. The Hoechst-positive nuclei were selected in P1. Further gating for size was applied (P2, P3) to remove doublets and aggregates. d. Example of a negative control with the same parameters as c. [file 41586_2020_2797_MOESM1_ESM.pdf]

nature research

https://doi.org/10.1038/s41586-020-2797-4

Supplementary information

Cells of the adult human heart

In the format provided by the authors and unedited

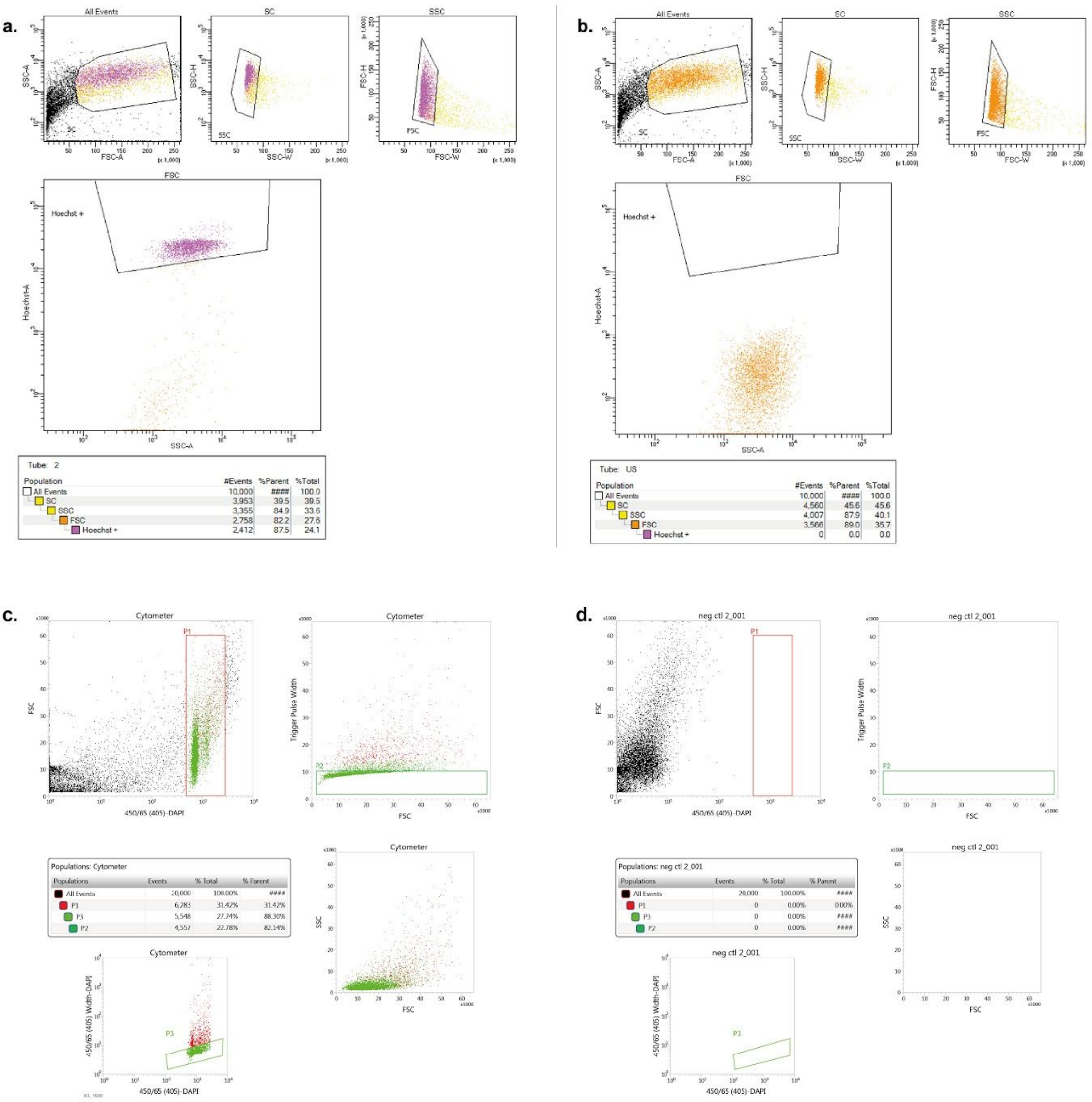

Supplementary Figure 1 - FACS gating strategy: Gating strategy for removal of cell debris and purification of nuclei. **a.** Size gating to remove doublets and aggregates was applied (SSC-A, FSC-A, SSC-W, SSC-H, FSC-H, FSC-W), followed by sorts for Hoechst-positive nuclei (purple population). Used for all nuclei processed in North America **b.** Example of negative control (no NucBlue Live ReadyProbes Reagent applied, ThermoFisher) with the same parameters as **a. c.** Gating strategy used for all nuclei processed in the United Kingdom. The Hoechst-positive nuclei were selected in P1. Further gating for size was applied (P2, P3) to remove doublets and aggregates. **d.** Example of a negative control with the same parameters as **c.**
