# Supplementary material for: Cells of the adult human heart
Source: Nature. 2020 Sep 24;588(7838):466–72. doi: 10.1038/s41586-020-2797-4 (PMC7681775; doi:10.1038/s41586-020-2797-4)
Supplement: Supplementary file 2 — Reporting Summary [file 41586_2020_2797_MOESM2_ESM.pdf]

nature research

Corresponding author(s): Jonatha Seidman, Christine E. Seidman, Michela Noseda, Norbert Hubner, Sarah A. Teichmann

Last updated by author(s): August 21st, 2020

# Reporting Summary

Nature Research wishes to improve the reproducibility of the work that we publish. This form provides structure for consistency and transparency in reporting. For further information on Nature Research policies, see our Editorial Policies and the Editorial Policy Checklist.

## Statistics

For all statistical analyses, confirm that the following items are present in the figure legend, table legend, main text, or Methods section.

| n/a | Confirmed | |
|---|---|---|
| ☐ | ☒ | The exact sample size ($n$) for each experimental group/condition, given as a discrete number and unit of measurement |
| ☐ | ☒ | A statement on whether measurements were taken from distinct samples or whether the same sample was measured repeatedly |
| ☐ | ☒ | The statistical test(s) used AND whether they are one- or two-sided<br>*Only common tests should be described solely by name; describe more complex techniques in the Methods section.* |
| ☐ | ☒ | A description of all covariates tested |
| ☐ | ☒ | A description of any assumptions or corrections, such as tests of normality and adjustment for multiple comparisons |
| ☐ | ☒ | A full description of the statistical parameters including central tendency (e.g. means) or other basic estimates (e.g. regression coefficient) AND variation (e.g. standard deviation) or associated estimates of uncertainty (e.g. confidence intervals) |
| ☐ | ☒ | For null hypothesis testing, the test statistic (e.g. $F$, $t$, $r$) with confidence intervals, effect sizes, degrees of freedom and $P$ value noted<br>*Give P values as exact values whenever suitable.* |
| ☒ | ☐ | For Bayesian analysis, information on the choice of priors and Markov chain Monte Carlo settings |
| ☒ | ☐ | For hierarchical and complex designs, identification of the appropriate level for tests and full reporting of outcomes |
| ☒ | ☐ | Estimates of effect sizes (e.g. Cohen's $d$, Pearson's $r$), indicating how they were calculated |

*Our web collection on statistics for biologists contains articles on many of the points above.*

## Software and code

Policy information about availability of computer code

| | |
|---|---|
| Data collection | Software used include: BD FACS Sortware 1.2.0.142 (BD Influx), Summit V5.4.0.16584 (BD XDP), BD FACSDIVA 6.1.3 (Aria), Axiovision 4.9.1 and ZEN 2.3 software (Zeiss), Harmony 4.9 (Perkin Elmer), Chromium Controller Firmware version 5.00 (10X Genomics). |
| Data analysis | Software used include: R 3.6, Python 3.7, 10X Genomics’ Cell Ranger 3.0.2, Harmony 4.9 (Perkin Elmer), Axiovision 4.9.1 and ZEN 2.3 (Zeiss), Adobe Illustrator 24.2.3, CorelDraw X4, 10X Genomics' Space Ranger 1.0.0, ImageJ 1.52Q or 1.52P, Scanpy 1.4, bbknn 1.3.11, scGen 6c237d7, anndata 1.7, pandas 1.0.1, numpy 1.19, scVelo 1d87464. |

For manuscripts utilizing custom algorithms or software that are central to the research but not yet described in published literature, software must be made available to editors and reviewers. We strongly encourage code deposition in a community repository (e.g. GitHub). See the Nature Research guidelines for submitting code & software for further information.

## Data

Policy information about availability of data

All manuscripts must include a data availability statement. This statement should provide the following information, where applicable:

- Accession codes, unique identifiers, or web links for publicly available datasets
- A list of figures that have associated raw data
- A description of any restrictions on data availability

The data is made available through the Human Cell Atlas (HCA) Data Coordination Platform (DCP) and can be accessed here: https://www.ebi.ac.uk/ena/browser/view/ERP123138. The data can also be accessed and explored through the HCA Heart Project website at www.heartcellatlas.org.

# Field-specific reporting

Please select the one below that is the best fit for your research. If you are not sure, read the appropriate sections before making your selection.

- [x] Life sciences
- [ ] Behavioural & social sciences
- [ ] Ecological, evolutionary & environmental sciences

For a reference copy of the document with all sections, see nature.com/documents/nr-reporting-summary-flat.pdf

# Life sciences study design

All studies must disclose on these points even when the disclosure is negative.

**Sample size**

No sample-size calculation was done due to the nature of this study.
Non-failing hearts were collected from human donors from July 2018 to July 2019 on the basis of availability from CBTM (Cambridge, UK) and The University of Alberta (Canada). Our study explores the cellular composition of the healthy adult human heart and we state that the number of samples is not enough to make generalisations.

**Data exclusions**

No data were excluded from the analysis. For the final count matrix, we excluded cells based on pre-established criteria for single-cells: we excluded low quality samples and contaminating cells (i.e. - cells with low number of detected genes and high mitochondria content).

**Replication**

We performed single nuclei RNAseq on 14 hearts (4 - 6 regions each) and single cell RNAseq on 7 hearts (4 - 6 regions each), with comparable results among all the donors. The same samples were used for the validation experiments. The micrographs in Figure 2g, 3c/h and Extended Data Figure 2c (HAMP), 2e (CNN1), 3f, 3k, 4f are repeated with similar results in 2 individual tissue sections. The micrographs in Figure 2h, 3f and Extended Data Figure 2c (CNN1), 2e (PCDH7), 4e, 4h, 6d are repeated with similar results in 3 individual tissue sections. The micrographs in Figure 1e, 2d, 3e, and Extended Data Figure 1f, 2c (FHL1) and 4d are repeated with similar results in 4 individual tissue sections. The micrographs in Figure 2c and Extended Data Figure 2c (PRELID2), 7e, 9a are repeated with similar results in 6 or more individual tissue sections. Positive and negative controls were done once per used samples.

For skeletal muscle analysis, single cells and single nuclei were isolated from 5 individuals, with comparable results among all the donors.

**Randomization**

Only healthy individuals were considered in our analysis. Randomisation was not relevant due to the study design where non-failing hearts were used on availability.

**Blinding**

Only healthy individuals were considered in our analysis. Blinding was not relevant due to the study design where non-failing hearts were used on availability, and the analytical strategy would not benefit from it.

# Reporting for specific materials, systems and methods

We require information from authors about some types of materials, experimental systems and methods used in many studies. Here, indicate whether each material, system or method listed is relevant to your study. If you are not sure if a list item applies to your research, read the appropriate section before selecting a response.

## Materials & experimental systems

| n/a | Involved in the study |
|---|---|
| ☐ | ☒ Antibodies |
| ☒ | ☐ Eukaryotic cell lines |
| ☒ | ☐ Palaeontology and archaeology |
| ☒ | ☐ Animals and other organisms |
| ☐ | ☒ Human research participants |
| ☒ | ☐ Clinical data |
| ☒ | ☐ Dual use research of concern |

## Methods

| n/a | Involved in the study |
|---|---|
| ☒ | ☐ ChIP-seq |
| ☐ | ☒ Flow cytometry |
| ☒ | ☐ MRI-based neuroimaging |

## Antibodies

**Antibodies used**

anti-human CD45 monoclonal antibody-conjugated microbeads (Miltenyi Biotec, 130-045-801) in dilution 1:4 (20 ul of antibody-labeled microbeads in 80 ul of cell suspension buffer).

**Validation**

Commercially available product, full protocol and validation available at miltenyibiotec.com/_Resources/Persistent/25cf8ecca93dc183f1d96d5348e58ca0e9a07c40/DS130-045-801.pdf

## Human research participants

Policy information about studies involving human research participants

**Population characteristics**

Tissues were obtained form 14 individuals, eight (D1-7 and 11) collected in the United Kingdom and six (H2-7) collected in

| | |
|---|---|
| Population characteristics | North America. The cohort consisted of seven male (D2, D3, D6, D7, H2, H3 and H4) and seven female (D1, D4, D5, D11, H5, H6 and H7) donors, in the range of 40-75 years of age. Six of the donors were classified as DCD (Donation after Circulatory Death, D2, D4-7 and D11) and eight donors were classified as DBD (Donation after Brain Death, D1, D3, H2-7). |
| Recruitment | Cardiovascular history was unremarkable for all donors, and this was the main recruitment criteria used for to include individuals in our study. We believe this method of recruitment does not represent any bias that can impact our results. |
| Ethics oversight | Heart tissues (D1-7 and 11) were obtained from deceased transplant organ donors after Research Ethics Committee approval (Ref 15/EE/0152, East of England - Cambridge South Research Ethics Committee) and informed consent from the donor families.<br><br>Heart tissues (H2-7) were obtained from deceased organ donors after Human Research Ethics Board approval Pro00011739 (University of Alberta, Edmonton, Canada). Informed consent from donor families was acquired via the institutional Human Organ Procurement and Exchange Program (HOPE). |

Note that full information on the approval of the study protocol must also be provided in the manuscript.

# Flow Cytometry

## Plots

Confirm that:

☒ The axis labels state the marker and fluorochrome used (e.g. CD4-FITC).

☒ The axis scales are clearly visible. Include numbers along axes only for bottom left plot of group (a 'group' is an analysis of identical markers).

☒ All plots are contour plots with outliers or pseudocolor plots.

☒ A numerical value for number of cells or percentage (with statistics) is provided.

## Methodology

| | |
|---|---|
| Sample preparation | As described in the Methods section. Briefly, the single nuclei were isolated by mechanical homogenisation and washed. The nuclei were stained with commercially available Hoechst 33342 dye (NucBlue , R376050). The samples were kept on ice and directly loaded onto the FACS-sorter. |
| Instrument | Becton Dickinson (BD) Influx, XDP, or FACSAria |
| Software | Proprietary software of the selected sorter. |
| Cell population abundance | N/A |
| Gating strategy | Single nuclei were selected for single signal on the SCC and FCC to avoid aggregates. The Hoechst-positive nuclei were selected without any size limit. The gating strategy is available as Supplementary Figure 1. |

☒ Tick this box to confirm that a figure exemplifying the gating strategy is provided in the Supplementary Information.
