# Supplementary material for: Cells of the adult human heart
Source: Nature. 2020 Sep 24;588(7838):466–72. doi: 10.1038/s41586-020-2797-4 (PMC7681775; doi:10.1038/s41586-020-2797-4)
Supplement: Supplementary file 3 — This zipped file contains Supplementary Tables 1-35 and their legends. [file 41586_2020_2797_MOESM4_ESM.zip › Supplementary_tables/Supplementary_Table_24.docx]

**METHODS KEY RESOURCES TABLE**

| **REAGENT or RESOURCE** | **SOURCE** | **IDENTIFIER** |
| --- | --- | --- |
| **Antibodies** |  |  |
| Anti-human CD45 monoclonal | Miltenyi Biotec | 130-045-801 |
| **Chemicals** |  |  |
| 10% formalin neutral buffered | Sigma-Aldrich | HT501128 |
| 1M HEPES | Invitrogen | 15630 |
| 1M MgCl2 | ThermoFisher | AM9530G |
| 1M Tris buffer, pH 8 | ThermoFisher | AM9855G |
| 2M KCl | ThermoFisher | AM9640G |
| 2-Methylbutane, 99+%, extra pure, Acros Organics (Isopentane) | ThermoFisher | 126470025 |
| 4% paraformaldehyde | ThermoFisher | AAJ19943K2 |
| 70% Ethanol | VWR | 20823.327 |
| Bovine Serum Albumin (BSA) | Sigma-Aldrich | A3059-50G |
| cOmplete™, EDTA-free Protease Inhibitor Cocktail | Merck | 11873580001 |
| DNaseI | Roche | 11284932 |
| dPBS | Gibco | 11419013 |
| Dithiothreitol (DTT) | ThermoFisher | P2325 |
| Fetal Bovine Serum (FBS) | Gibco | 11284932 |
| GentleMACS C-tube | Miltenyi Biotec | 130-093-237 |
| HBSS | Invitrogen | 2420-117 |
| Liberase TH Research Grade | Roche | 5401151001 |
| NucBlue | ThermoFisher | R37605 |
| Nuclease-Free Water | Sigma-Aldrich | W4502 |
| O.C.T. Compound, Tissue-Tek, Sakura Finetek | VWR | 25608-930 |
| Protector RNAseIn 40 U/μl | Merck | 3335402001 |
| RNasin Plus 40 U/μl | Promega | N2611 |
| Sucrose | Sigma-Aldrich | S0389 |
| SuperaseIn 20 U/μl | ThermoFisher | AM2696 |
| Taurin | Sigma-Aldrich | T8691 |
| Triton X-100 10% (v/v) | Sigma-Aldrich | T8787 |
| Collagenase, type 2 | Worthington-Biochem | LS0004177 |
| Dispase | Sigma-Aldrich | D4818 |
| Percoll | Sigma-Aldrich | P1644 |
| **Tools** |  |  |
| Dounce tissue grinder set | Merck | D9063 |
| Scissors | FST | 14518-14 |
| SuperFrost slides | ThermoFisher | 10149870 |
| **Critical Commercial Assays** |  |  |
| Bioanalyzer High Sensitivity DNA Analysis | Agilent | 5067-4626 |
| 4200 TapeStation System | Agilent | G2991AA |
| Chromium Single Cell 3′ Gel Bead and Library Kit | 10X Genomics | 120235, 120234, 120236, 120262 |
| Chromium Next GEM Single Cell 3′ Gel Bead and Library Kit v2 and v3 | 10X Genomics | 1000128, 1000127, 1000157 |
| RNAScope Multiplex Fluorescent Assay | ACDBio | 320850 |
| RNAScope Multiplex Fluorescent v2 Assay | ACDBio | 323100 |
| **Software and Algorithms/machine** |  |  |
| XDP or FACSAria™ | BD Biosciences |  |
| GentleMACS Octo Dissociator | Miltenyi Biotec |  |
| HiSeq4000 | Illumina |  |
| Nextseq500 | Illumina |  |
| LSM710 confocal microscope | Zeiss |  |
| Opera Phenix High-Content confocal Screening System | Perkin Elmer |  |
