# Supplementary material for: Cells of the adult human heart
Source: Nature. 2020 Sep 24;588(7838):466–72. doi: 10.1038/s41586-020-2797-4 (PMC7681775; doi:10.1038/s41586-020-2797-4)
Supplement: Supplementary file 3 — This zipped file contains Supplementary Tables 1-35 and their legends. [file 41586_2020_2797_MOESM4_ESM.zip › Supplementary_tables/Supplementary_Table_27.docx]

**Supplementary Table H1**: *GWAS studies used for magma analyses*.

| **First author** | **PMID** | **prefix** | **Trait** |
| --- | --- | --- | --- |
| Roselli | 29892015 | AF_GWAS | Atrial Fibrillation |
| Nelson | 28714975 | CAD_GWAS | Coronary artery disease |
| Shah | NA | HF_GWAS | Heart failure |
| Aragam | 30586722 | HF_UKBB | Heart failure |
| Aragam | 30586722 | NICM_UKBB | Non-ischemic cardiomyopathy |
| Lin | 29748316 | PR_GWAS | PR interval |
| Christophersen | 28794112 | PWAVE_GWAS | P wave duration |
| Prins | 30012220 | QRS_GWAS | QRS interval |
| Watanabe | 31427789 | HT_UKBB | Hypertension |
| Kanai | 29403010 | LVD_JPBB | Left ventricular diameter |
| Mahajan | 30297969 | T2D_GWAS | Type 2 diabetes |
| Wojcik | 31217584 | QT_GWAS | QT interval |
| den Hoed | 23583979 | HR_GWAS | Heart rate |
