# Supplementary material for: Cells of the adult human heart
Source: Nature. 2020 Sep 24;588(7838):466–72. doi: 10.1038/s41586-020-2797-4 (PMC7681775; doi:10.1038/s41586-020-2797-4)
Supplement: Supplementary file 3 — This zipped file contains Supplementary Tables 1-35 and their legends. [file 41586_2020_2797_MOESM4_ESM.zip › Supplementary_tables/HCA_Heart_Legends_for_supplementary_tables_200727.docx]

**Legends for all supplementary tables:**

**Supplementary Table 1**: Table with all available donor covariates metadata

**Supplementary Table 2**: Distribution of the eleven major cardiac cell types across the six sampled regions of the heart. n=14 biologically independent donors, 6 cardiac regions

**Supplementary Table 3**: Table of correlation in proportions between cell types. Linear regression models (correlations) are obtained using the R linear model function (lm), which estimates statistical likelihood (p-value) of a linear relationship. Bonferroni correction is applied for multiple testing. n=14 biologically independent donors, 6 cardiac regions

**Supplementary Table 4**: Average gene expression across cardiomyocyte subtypes and region

**Supplementary Table 5**: Cardiomyocyte population distribution by region. n=14 biologically independent donors. 6 cardiac regions. Student t-tests (t-tests) are used to compare cell type distributions at each site

**Supplementary Table 6**: Differentially expressed genes per cardiomyocyte population calculated using a Wilcoxon Rank Sum Test with Benjamini-Hochberg correction

**Supplementary Table 7**: Differential expression of cardiomyocyte genes per region calculated using a Wilcoxon Rank Sum Test with Benjamini-Hochberg correction

**Supplementary Table 8**: Differentially expressed genes for vascular cell states calculated using a Wilcoxon Rank Sum Test with Benjamini-Hochberg correction

**Supplementary Table 9**: Distribution of vascular cell states per region and per source

**Supplementary Table 10**: Cell-cell interactions between vascular cells in the heart using CellPhoneDB statistical inference

**Supplementary Table 11**: Differentially expressed genes for fibroblasts calculated using a Wilcoxon Rank Sum Test with Benjamini-Hochberg correction

**Supplementary Table 12**: Genes associated to the Oncostatin M pathway

**Supplementary Table 13**: Differentially expressed genes for atrial and ventricular fibroblasts calculated using a Wilcoxon Rank Sum Test with Benjamini-Hochberg correction

**Supplementary Table 14**: Distribution of immune cells across different data sources

**Supplementary Table 15**: Differentially expressed genes for cardiac immune populations calculated using a Wilcoxon Rank Sum Test with Benjamini-Hochberg correction

**Supplementary Table 16**: Number of predicted cardiac cells in skeletal muscle and kidney using logistic regression

**Supplementary Table 17**: Cell-cell interactions between human cardiac immune cells, cardiomyocytes and fibroblasts using CellPhoneDB statistical inference on 69 295 cardiomyocytes, fibroblasts and myeloid cells from 14 donors (n = 14)

**Supplementary Table 18**: Cell-cell interactions between human skeletal muscle immune cells, myocytes and fibroblasts using CellPhoneDB statistical inference

**Supplementary Table 19**: Transcriptional signatures from external studies for immune tissue residency and yolk sac derived macrophages

**Supplementary Table 20**: Differentially expressed genes in cardiac neuronal cells calculated using a WIlcoxon Rank Sum Test with Benjamini-Hochberg correction

**Supplementary Table 21**: Differentially expressed genes for cardiac adipocytes calculated using a Wilcoxon Rank Sum Test with Benjamini-Hochberg correction

**Supplementary Table 22**: Table with all available donor metadata, including age, gender, data source and sample id

**Supplementary Table 23**: List of probes used in smFISH experiments

**Supplementary Table 24**: List of reagents used in this study

**Supplementary Table 25**: Source data for the GO term analysis of the vascular cells. Differential expressed genes for each cell type was used as input for gene set enrichment analysis using a hypergeometric distribution as implemented in ToppFun

**Supplementary Table 26**: Source data for the GO term analysis of the adipocytes. Differential expressed genes for each cell type was used as input for gene set enrichment analysis using a hypergeometric distribution as implemented in ToppFun

**Supplementary Table 27**: GWAS studies used for MAGMA analysis

**Supplementary Table 28**: Source data from the MAGMA output for the visualisation of GWAS hits in the cardiac cell types

**Supplementary Table 29**: Distribution of cell types per data source after subclustering analyses with cell type-specific cutoffs. n=14 biologically independent donors. 6 cardiac regions

**Supplementary Table 30**: Table of cell and nuclei dispersion per isolation method, excluding cardiomyocytes. n=14 biologically independent donors. 6 cardiac regions

**Supplementary Table 31**: Differentially expressed genes for human skeletal muscle cell populations calculated using a Wilcoxon Rank Sum Test with Benjamini-Hochberg correction

**Supplementary Table 32**: Differentially expressed genes for vascular cells of the human skeletal muscle calculated using a Wilcoxon Rank Sum Test with Benjamini-Hochberg correction

**Supplementary Table 33**: Cell-cell interactions for vascular cells of the human skeletal muscle. Using CellPhoneDB statistical inference, we calculated cell-cell interactions with 9 220 cells from five donors (n = 5)

**Supplementary Table 34**: List of gene symbols used in this study

**Supplementary Table 35**: Distribution of fibroblasts cell states per region
